# The evolving topology of the Lightning Network: Centralization, efficiency, robustness, synchronization, and anonymity

**DOI:** 10.1371/journal.pone.0225966

**Published:** 2020-01-15

**Authors:** Stefano Martinazzi, Andrea Flori

**Affiliations:** Politecnico di Milano, Department of Management, Economics and Industrial Engineering, Milan, Italy; Universita Cattolica del Sacro Cuore, ITALY

## Abstract

The Lightning Network (LN) was released on Bitcoin’s mainnet in January 2018 as a solution to favor scalability. This work analyses the evolution of the LN during its first year of existence in order to assess its impact over some of the core fundamentals of Bitcoin, such as: node centralization, resilience against attacks and disruptions, anonymity of users, autonomous coordination of its members. Using a network theory approach, we find that the LN represents a centralized configuration with few highly active nodes playing as hubs in that system. We show that the removal of these central nodes is likely to generate a remarkable drop in the LN’s efficiency, while the network appears robust to random disruptions. In addition, we observe that improvements in efficiency during the sample period are primarily due to the increase in the capacity installed on the channels, while nodes’ synchronization does not emerge as a distinctive feature of the LN. Finally, the analysis of the structure of the network suggests a good preservation of nodes’ identity against attackers with prior knowledge about topological characteristics of their targets, but also that LN is probably weak against attackers that are within the system.

## Introduction

Since its inception, Bitcoin has been known as a technology unable to perform a great amount of transactions per unit of time [1]. Being coded in such a way that on average a single block is mined and added to the blockchain every ten minutes, Bitcoin can perform a maximum of seven transactions per second. In comparison, Visa can routinely process two thousand transactions per second, with peaks of several thousand transfers [1, 2].

Miners are those players in this system that can build and add new constituencies to the blockchain, so putting them in place to impose higher fees in times of great demand. The most emblematic example occurred in 2017, when fees skyrocketed from less than $1 per transaction to a maximum of nearly $40 [3]. Fees mainly depend on the amount of transactions waiting to be added in the blockchain, regardless of the volume of Bitcoins transacted per time. For large transferred amounts, the blockchain can therefore be very cheap compared to traditional means of payment, potentially moving the equivalent of several million of dollars for only a few cents, while it can be extremely economically inefficient for routine payments and for micro-payments. These aspects contribute to stimulate the growing interest for the deployment of blockchain solutions in financial applications [4–6].

It is against this background that some attempts have been proposed to increase throughput and lower latencies. For instance, a hardfork of the Bitcoin’s blockchain occurred in November 2017 with the implementation of the Segregated Witness (SegWit, Bitcoin Improvement Proposal 141) that quadruplicated the number of transactions that can be placed into a single block. Another example occurred in August 2017 with the hardfork that created Bitcoin Cash, a version of Bitcoin with blocks of 8Mb.

Among these infrastructural improvements, a recent novelty refers to the deployment of the Lightning Network (hereinafter, LN). LN was initially proposed on February 2015, while the corresponding mainnet was launched in January 2018, after a period of testing on a copy of the original Bitcoin’s blockchain called “Testned”. LN is a system of channels for micro-payments built on top of Bitcoin’s blockchain and, therefore, indicated as a “Layer 2” solution based on smart contracts. In practice, two counterparts can decide to open a bilateral channel by issuing a multi-signed transaction on the blockchain, thereafter, allowing them to exchange back and forth a predefined amount of bitcoins. This system is based on off-chain transactions, which means that transactions on the LN do not need to be uploaded on the blockchain at each iteration [7]. Eventually, a multi-signed transaction corresponding to the final balance between the two counterparts will be released to the blockchain when that channel is no longer needed. For this reason, nowadays LN is considered among the most recognized solutions for scalability.

**Fig 1:**
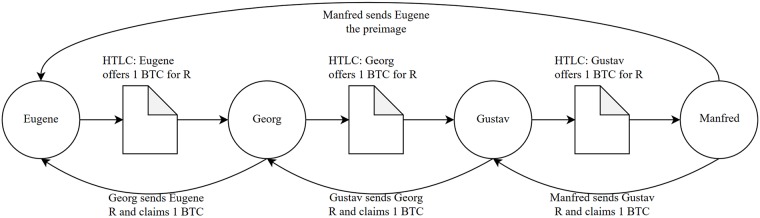
Representation of a multi-hop transaction.

More practically, to open a channel in the LN, a preliminary transaction (namely, the “channel funding”) between two counterparts is issued on the blockchain. After that initial transaction, these two counterparts need to issue new “commitment transactions” in order to exchange additional flows. These transactions simply refer to the balance of the channel signed by the two counterparts, whose amount is not required to be broadcasted to the entire network. The only exception is the final commitment, also referred to as a “closing transaction”, since it closes the bilateral channel and sets the new balance on the blockchain. If a channel is excessively unbalanced towards one counterpart, then this channel is considered “unbalanced” and can constitute a problem for other peers interested in exploiting that edge to route their transactions. This multi-hop framework allows one party to send payments to other counterparts, without issuing a brand-new channel, whenever a common path linking more channels is present and has enough available capacity. As shown in Fig 1, if node “Eugene” wants to send one Bitcoin to node “Manfred” on the LN without opening a direct channel, it must search for another node that is connected to both these sources and target nodes with enough capacity to allow the transfer of one Bitcoin to “Manfred” in exchange of one Bitcoin from “Eugene” plus a fee. This mechanism is based on Hashed Time Lock Contracts (HTLCs), which are cryptographic agreements issued off-chain and utilized to make it extremely difficult for nodes in the multi-hop path to steal the amount transacted through them [8, 9]. In the illustrative example presented in Fig 1, nodes “Georg” and “Gustav” represent those intermediate nodes through which the multi-hop transaction can be performed and connect “Eugene” with “Manfred”.

The interest around LN and its promises for a scalable use of Bitcoin lead many to invest time and money in its development and implementation. One year after its inception on the mainnet, we believe it is time to assess the performance of the LN along some of the features that motivated its deployment. For instance, during the development of the LN, one of the most concerning aspects has been the possibility that some participants would become very central in that system. This issue resides in the nature of the multi-hop framework. Counterparts with higher capacity are, in fact, more likely to act as payment hubs, *de facto* centralizing the underlying system [1]. The centralization of the LN would create several concerns about its functioning and privacy. Hubs may collect, in fact, information on a huge number of counterparts and even censor transactions or raise fees thanks to their key position in the system [10].

To gauge such emerging topological features, we perform a network analysis of the LN using one year data from the launch of the LN at the beginning of 2018 to January 2019. We note a tendency towards a centralized structure with a few highly connected nodes. This aspect could pose a threat and a drawback for the value propositions of Bitcoin. Highly connected nodes could be used, in fact, to harvest a great amount of information coming from the flow they intercept. This means that even if the sending node changes the routing plan, then there is still a high probability that such central nodes, playing as hubs, are so well connected to the rest of the system to be included again in the alternative new path. Even if the hub is legit, its presence could therefore constitute an issue for the functioning of the LN and its adoption.

The identification of the topological properties of the LN has, therefore, guided our assessment of its performance. For instance, very central nodes could pose as preferential targets for attacks perpetrated to destabilize the network. We notice, in fact, that the removal of key central nodes are likely to determine a disruptive effect, while the network shows a remarkable robustness against random failures. Interestingly, we also note that during the sample period, the efficiency of the LN has shown an overall increase in its ability to transfer information mainly due to the growth in the number of edges and their stored capacities rather than their better allocation within the network. We also tackle the issue of synchronization among nodes, which is an aspect strictly related to the efficiency of the network. We envisage each edge as a binary oscillator, from an open to a close position representing the state of the balance of the channel connecting two counterparts. The absence of coordination in the way channels are re-balanced may, in fact, limit the overall adoption of the underlying infrastructure. Our analysis reveals a slight deterioration of the network’s capability to promote coordination in the way participants open and close their channels during the sampled period. Finally, we assess the anonymity extent of the LN, which is another key feature of the Bitcoin framework and we find that our estimates depict a LN which is becoming more capable to protect users’ identity from attackers outside the system, while it appears less able to preserve anonymity from inner attackers.

## Materials and methods

To study how the LN has evolved during the sample period, we follow similar approaches proposed by [11–13] for the Bitcoin’s transaction graph, thus adopting a network perspective where each node is a single address representing a user. Edges between pairs of nodes are, instead, the actual channels created by issuing a transaction on the blockchain, while their capacity is measured by the amount of stored Bitcoins (hereinafter, BTC).

Our reference period ranges over an entire year from the 12th of January 2018, which corresponds to the launch of the LN on the mainnet, to the 12th of January 2019. Our final dataset is comprised of about 4189 different nodes involved in 67917 channels. We describe the latter by the pairs of nodes involved in the respective channels, the opening and closing dates (if the channels have been closed during the sample period), the amount of stored BTC and the corresponding value converted in USD.

We employ the reciprocal of the capacity of the nodes to create an undirected weighted network. The unweighted version of the LN would provide an inaccurate representation of the system since it poses poorly endowed edges with the same capability to perform the multi-hop routing as those edges richer in terms of stored BTC. This aspect is particularly relevant for practical purposes as highlighted in [14], where it has been shown that the probability to successfully route a payment drops dramatically for values above a few dollars.

For representative purposes, the dataset has been divided into twelve snapshots corresponding to the twelfth of each month from February 2018 to January 2019. Although such investigation framework would prevent a proper analysis of the time dynamics governing the evolution of the LN, it allows us to depict the main features and their changes in time that are at the ground level of the core fundamentals of the phenomenon under study. We provide some descriptive topological properties of these twelve snapshots in Table 1.

**Table 1:** A collection of topological measures for the LN. Columns in the table refer respectively to: number of nodes, number of edges, density of the network, median degree, median strength, average degree, average strength, average edges’ capacity, total capacity of the network, diameter, radius, transitivity, portion of the capacity of the edges composing the minimum spanning tree, assortativity coefficients for both the weighted and unweighted networks, correlation between nodes’ degree and their average capacity (asterisks **,*** refer to significance at 1% and 0.1%, respectively). We refer to the weighted adjacency matrix as *W*. Strength and capacity are expressed in USD.

	**Nodes**	**Edges**	**Density**	**Median** **Degree**	**Median** **Strength**	**Avg.** **Degree**	**Avg.** **Strength**	**Avg. Edge** **Capacity**
**Feb-18**	518	1910	0.014	2	22.09	7.33	208.77	28.31
**Mar-18**	733	2060	0.008	2	18.91	5.60	121.15	21.56
**Apr-18**	1359	6029	0.006	3	14.71	8.70	161.89	18.25
**May-18**	1721	8172	0.005	3	17.72	9.35	203.95	21.48
**Jun-18**	1808	7876	0.005	3	13.78	8.57	174.18	19.99
**Jul-18**	2039	8996	0.004	3	15.01	8.57	380.66	43.14
**Aug-18**	2130	11137	0.005	3	21.80	10.07	564.55	53.99
**Sep-18**	2337	12312	0.004	3	25.50	10.01	621.25	58.96
**Oct-18**	2466	12429	0.004	3	30.54	9.62	578.33	57.37
**Nov-18**	2626	12958	0.004	3	31.33	9.47	558.71	56.61
**Dec-18**	2878	17086	0.004	3	20.90	11.40	1136.71	95.73
**Jan-19**	3613	23853	0.003	3	33.65	12.48	1173.98	88.91
	**Total** **Capacity ($)**	**Diameter**	**Radius** **(LCC)**	**Transitivity** **(W)**	**MST** **(W)**	**Assortivity** **(W)**	**Assortivity**	**Degree-Strength** **correlation**
**Feb-18**	54072	6	4	12%	66%	-0.16	-0.37	0.03
**Mar-18**	44401	7	4	5%	74%	-0.14	-0.37	0.02
**Apr-18**	110003	7	4	9%	68%	-0.05	-0.27	0.03
**May-18**	175503	8	5	9%	64%	-0.06	-0.29	0.04
**Jun-18**	157455	8	5	7%	64%	-0.05	-0.28	0.04
**Jul-18**	388082	8	5	7%	69%	-0.01	-0.26	0.05 **
**Aug-18**	601241	8	5	9%	55%	-0.03	-0.25	0.14***
**Sep-18**	725934	8	5	9%	48%	-0.07	-0.26	0.17***
**Oct-18**	713085	8	5	9%	46%	-0.07	-0.25	0.16***
**Nov-18**	733584	9	5	8%	48%	-0.07	-0.27	0.14***
**Dec-18**	1635724	9	5	10%	25%	0.01	-0.24	0.25***
**Jan-19**	2120788	9	5	10%	25%	-0.07	-0.22	0.21***

The largest connected components for each of these snapshots account for almost the entire network, with only a few disconnected components mainly composed by single pairs. The number of nodes simultaneously on-line in our time snapshots grows from 518 (in February 2018) to 3613 (in January 2019), while the corresponding number of channels increases from 1910 to 23853. This determines a decreasing pattern in the density of the links present in the network, which is only 1.45% in February 2018 and reaches even lower values in January 2019 (about 0.37%). The LN has been evolving, therefore, from a fairy sparse initial configuration to even higher levels of sparsity along its short life. Interestingly, the degree distribution shows the tendency of the network to establish a few channels per node. The median degree, for instance, increases from a value of only 2 (in February 2018) to 3 (in January 2019) edges per node, while the corresponding average values move from about 7 to 12.5. This is an interesting aspect of the LN given its need to route transactions, but also given the vocation of the Bitcoin framework to be an uncentralized system.

However, an important aspect is the distribution of the strength and its evolution. Here we refer to the strength of a node as determined by the weighted sum of all its edges, taking into consideration the fact that nodes with higher values of strength stand for users with higher capability to accept flows of transactions through their channels. The median value stays almost stable over time (ranging between $13.78 and $33.65), while the average value quintuplicates during the sample period (from $208.77 in the first observation to $1173.98 in the last one). This clearly signals the enlargement of the network and, possibly, the deployment of very active nodes. Similarly, the average capacity installed on the channels increased considerably. As a result, the overall total capacity of the system exhibits a sharp increase during the sample period.

Moreover, we explore the assortativity of the weighted network [15] and we find a slightly disassortative tendency along the entire period, thus placing the LN in analogy with infrastructural networks, such as railway stations [16], national airport systems [17], and information, technological and biological networks [18]. This negative relationship is emphasized in the unweighted version of the network. Surprisingly, we notice, however, how being highly connected with the rest of the system is not strongly correlated with the average capacity. This phenomenon could be in part explained by the different behaviors of “poor” vs. “wealthy” (in terms of their actual disposable BTC) nodes to form channels: “poor” nodes may opt to connect to hubs in order to save the transaction fees required to open and close channels, while more “wealthy” nodes may simply connect directly to other nodes bypassing hubs. In addition, many channels may have been created as an attempt to test the LN without committing too many *satoshis* (namely, this is the minimum amount of transferable BTC corresponding to 0.00000001 BTC). Finally, Table 1 shows that the portion of the capacity installed on edges that are part of the minimum spanning tree (MST) is decreasing over time, while the presence of simple patterns in the formation of edges (see, for instance, the Transitivity coefficient) has remained relatively stable. Although the network is expanding (see also the Diameter and the Radius coefficients), we thus observe that local structures appear diffused and recurrent over time.

## Results

The way nodes tend to create channels is of utmost importance for the goals of the LN to serve as a facilitating environment to favour scalability and adoption. The following sections will focus, therefore, on specific topological aspects directly connected to relevant pillars raised by the deployment of the LN. Firstly, we analyze the extent of centralization in the network, i.e., whether the network presents very central nodes that, playing a role like hubs, disobey the decentralization mission of the Bitcoin framework. Secondly, we assess the efficiency of the LN, i.e., its capacity to disseminate information through its nodes, which is a critical aspect for routing transactions. Thirdly, we focus on the robustness of the network, i.e., its resilience against multiple failures among its nodes that may occur due to hacking activities or infrastructural disruptions. Next, we study the synchronization level of the LN, an issue related to the possibility that multiple critical nodes may act with autonomous coordination, for instance by closing channels and damaging the overall efficiency of the network. Finally, we analyze the level of anonymity that is provided by the emerging network configuration.

### Centralization

One of the main concerns related to the LN refers to the emergence of configurations with very central nodes acting as hubs, thus undermining the Blockchain aim of promoting a highly decentralized system. In fact, since its establishment the LN has shown the presence of some very central players in terms of number of connections. However, although a binary representation is well diffused in network analysis, the LN is not, in practice, a binary system and the amount of capacity installed on each edge is of utmost importance for its functioning and scalability. Hence, simply referring to the degree distribution would basically mean that each channel is assumed to be identical, implying that those with a capacity of few satoshis are considered as important as those with a whole stored BTC, which are instead much more able to perform multi-hop transactions. For this reason, in this subsection we refer to the strength distribution, which has been already applied to characterize nodes’ centralities in many different contexts, such as stock markets, national railways and proteins [19–23].

**Fig 2:**
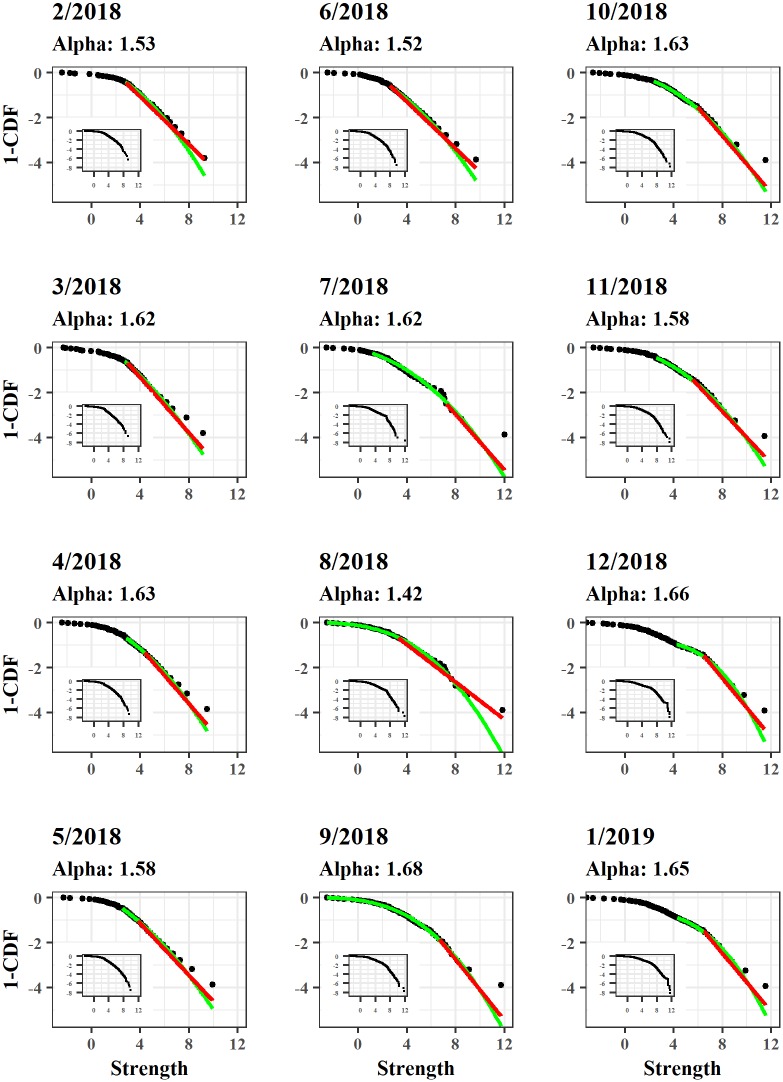
Strength distributions. The log-log plots show the fitted Power-Law (in red) and the Log-Normal (in green) distributions for the cCDF of the strength distribution. We binned data using 50 quantiles; to take into consideration the skeweness within the bins we aggregated by medians. The strength distributions for the original data are reported in the plot inserts. In December 2018 and January 2019 we can notice the sudden decay due to the limit in capacities embedded in the protocol.

To take into account the relevance of the capacity installed on the channels, we plot in Fig 2 the complementary cumulative distribution function of the strength values. We also visualize the fitted distribution of the strength against the Log-Normal (in green) and the Power-Law (in red) distributions. The latter is also tested with the variant of the Kolmogorov-Smirnov test proposed in [24]. For instance, a typical feature of a scale-free network, hence of a network with some very central nodes surrounded by a large cloud of more peripheral nodes, is the presence of a Power-Law like decline in the tail of the distribution [25, 26]. Indeed, as shown in Fig 2, the Power-Law seems to provide a reasonable fit along the sample period. Interestingly, the last two snapshots show also the presence of an exponential decay in the upper tail which is likely to be due to a technological constraint in the LN, given that the protocol itself limits the possible amount installed on a single channel to 2^24^ satoshis [27]. More generally, Fig 2 suggests that a bundle of nodes can be highly connected to the rest of the system, largely characterized by nodes with only a few weak (in terms of capacity) connections.

**Fig 3:**
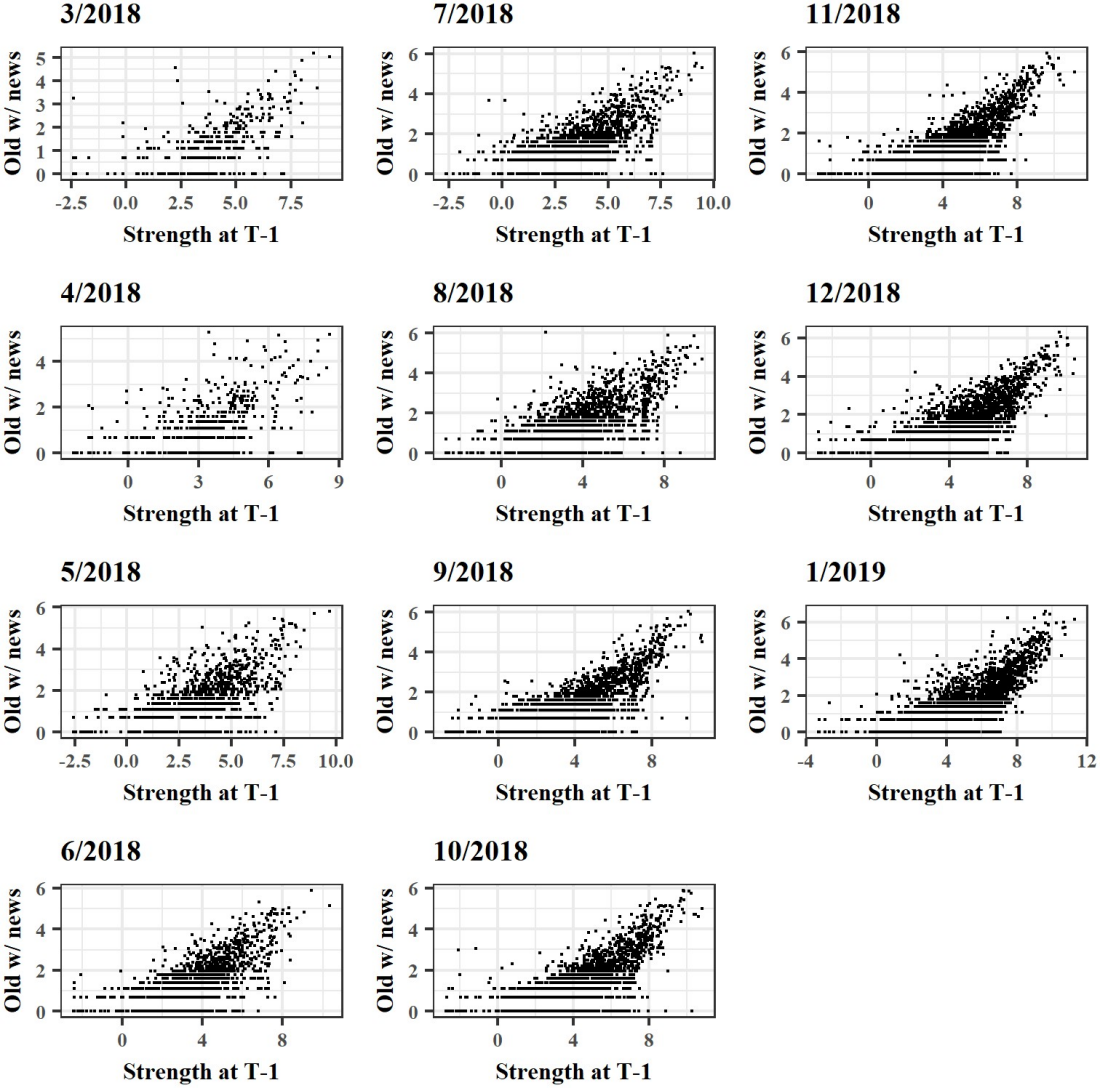
Tendency of incoming nodes to form channels. The log-log plots show the amount of channels formed by new nodes with well-established ones, the latter ranked on the *x*-axis with respect to their strength values at time *T* − 1.

The presence of hubs is also a key element to differentiate between random and scale-free networks. In many real-world cases, incoming nodes prefer in fact to create connections with already well-established ones [26, 28, 29]. Fig 3 shows the amount of connections between new nodes (spawned at time *T*) and the ones already present at time *T* − 1 versus the strength of the latter ones at time *T* − 1. A clear tendency for new nodes to prefer opening channels with already well-established nodes emerges from the figure. The LN seems that may resemble, therefore, an “hub and spoke” configuration with some extremely well connected and endowed nodes acting as hubs capable to attract and create connections with a great number of other new nodes.

We also notice the tendency of the network towards a more stable composition over time of the top wealthiest nodes in terms of capacity. For instance, among the 409 nodes that belong to the “top 5%” at least once in the time snapshots, only 62 appear more than 50% of the times. If we consider more recent observations, this proportion increases significantly since 138 out of 185 nodes are present in both December 2018 and January 2019. Moreover, the sample period witnessed a massive increase in the heterogeneity level of the strength distribution. In the first snapshot, the lower 5% percentile had an average strength of $0.2, compared to the top 5% that had average strength of about $2705. Conversely, at the end of the period such gap increased enormously, with the bottom 5% showing an average strength of $0.1 and the top 5% of about $17356.

Overall, these findings seem to support one of the original criticism regarding the capacity of the LN to remain a decentralized system. One may argue, in fact, that such emerging configuration is influenced by the multi-hop framework in which nodes, and in particular newcomers, have opted to form connections with few very central peers in order to efficiently spread transactions throughout the system. For this reason, in the next subsection we analyze whether the configuration of the system is able to effectively spread flows across its nodes by showing how the enlargement of the system has affected the evolution of the LN’s efficiency.

### Efficiency

A critical aspect for the functioning of the LN refers to the manner in which transactions are performed in the multi-hop framework. To study the efficiency of the network we employ the global efficiency of the network [30, 31] that measures the sum of the inverse of all the shortest paths of each node and normalizes it by the total number of possible connections. In a weighted graph, global efficiency is thus affected by both the level of interconnectivity between nodes and the distribution of the installed capacity among the edges. In formula: $E(G)=\frac{1}{N(N-1)}*\sum_{i\neq j\epsilon G}\frac{1}{d_{ij}}$, where *N* is the number of nodes in graph *G* and *d*_*ij*_ is the geodesic distance between *i* and *j*. Then, to better investigate the dynamics of the efficiency scores we normalize *E*(*G*) by the global efficiency of an ideal network of the same size that is completely connected and equally weighted (namely, *E*(*G*^*ideal*^)). In formula: $E_{Norm}\left(G\right)=\frac{E(G)}{E(G^{ideal})}$. These indicators help us to show how information (in our case Bitcoins) can move efficiently through the LN and reach different nodes. Hence, the higher the values of *E*(*G*) (or of *E*_*Norm*_(*G*)), the more efficient is the network.

A rise in the efficiency of a network can thus be due to the optimization of its structure, or due to an increase in the deployed resources across edges. Table 2 shows that the LN seems to have become more efficient over time as indicated by *E*(*G*), although when we consider *E*_*Norm*_(*G*) such improvement seems to be much more narrow. In addition, Table 2 reports the average local efficiency among all the nodes within the network (< *E*_*Local*(*G*) >), which is a measure of the efficiency of a node’s neighbourhood when deprived of that node. Interestingly, both the average local efficiency and its normalized variant (< *E*_*Local*_*Norm*_(*G*) >) indicate a trend fairly similar to the corresponding global efficiencies. Overall, the LN seems to be therefore a system that is gradually becoming more efficient, especially after the second half of 2018.

**Table 2:** Efficiency of the LN. Global and local efficiencies and their normalized variants against an ideal complete network where the total capacity is allocated evenly among all the *N*(*N* − 1) edges.

	Feb-18	Mar-18	Apr-18	May-18	Jun-18	Jul-18	Aug-18	Sep-18	Oct-18	Nov-18	Dec-18	Jan-19
*E*(*G*)	8.00	4.76	4.99	5.95	5.05	10.94	14.42	15.86	14.38	14.34	16.63	17.90
*E*_*Norm*_(*G*)	0.15	0.08	0.07	0.06	0.05	0.09	0.11	0.09	0.08	0.07	0.12	0.08
< *E*_*Local*(*G*) >	13.23	4.15	6.47	8.37	9.61	17.68	33.02	42.47	34.91	27.38	38.22	41.27
< *E*_*Local*_*Norm*_(*G*) >	0.25	0.07	0.10	0.08	0.10	0.15	0.24	0.25	0.18	0.13	0.27	0.18

There should exist, therefore, a trade-off between the efficiency of the ideal network and the actual cost of implementing it. Following [30], we take into account this aspect and we compute the network’s cost function as: $C_{Norm}\left(G\right)=\frac{\sum_{i\neq j\epsilon G}a_{ij}\gamma \left(l_{ij}\right)}{\sum_{i\neq j\epsilon G^{ideal}}\gamma \left(l_{ij}\right)}$, with *γ* the *cost evaluator* function that we assume to be linear (as in most of the cases studied in [30]), and entry *a*_*ij*_ equal to 1 if there is a link connecting node *i* to node *j*, and 0 otherwise. Hence, *C*_*Norm*_(*G*) assumes values in [0, 1], with 1 if the network is completely connected. In our case, the cost of opening a channel does not depend on other variables besides Bitcoin’s transaction fees, which we assume to be the same for each possible channel (i.e., the cost to connect node *i* to node *j* is assumed to be equivalent to open a channel between any other pair of nodes *x* and *y* in the network, regardless their installed capacities). For this reason, in this simplified case *C*_*Norm*_(*LN*) is equal to the density of the graph. As argued by [30], an “Economic Small World” (ESW) network presents both high global and local efficiencies at a low price once normalized against the ideal network. Comparing estimates of the efficiency of the World Wide Web and the Internet Network provided in [30], we notice that both of these networks have higher efficiency levels at lower prices than the LN. From this evidence, we can not yet refer to the LN as an ESW network. Low levels of efficiency and their relatively high cost may thus pose a challenge for the usage of the LN. If efficiency is too low, then the multi-hop routing system may no longer be able to connect nodes that do not share a direct channel, thus questioning the usefulness of the LN as an effective solution for scaling Bitcoin.

### Robustness

In the section about centralization, we have shown that the LN seems to present a power-law tail in its strength distribution, which is a common characteristic of scale-free networks. Scale-free networks are known to be resilient against random failures, but also to be very exposed to targeted attacks. For this reason they are often referred as a “robust yet fragile” configuration [32]. Moreover, [33] notice that many complex systems are resistant to drastic node removal by random failures or attacks and show how communication networks are surprisingly robust typically due to redundant wiring.

The presence of channels with different capacities implies that the LN resembles an infrastructure network. Taking into consideration only the unweighted topology of the network would possibly lead, therefore, to erroneous conclusions about its robustness and capability to withstand an aggression or disruption. This aspect has been discussed in a recent paper by [34], whose framework inspired our analysis. To evaluate the effective impact of nodes removal, following [34], we also monitor the variation in the size of the Largest Connected Component (LCC) after every round of nodes removal. Finally, in line with [35], we also track the variation of the average local efficiency, which is an alternative indicator to the clustering coefficient in measuring the fault tolerance of disconnected networks [30, 31].

**Fig 4:**
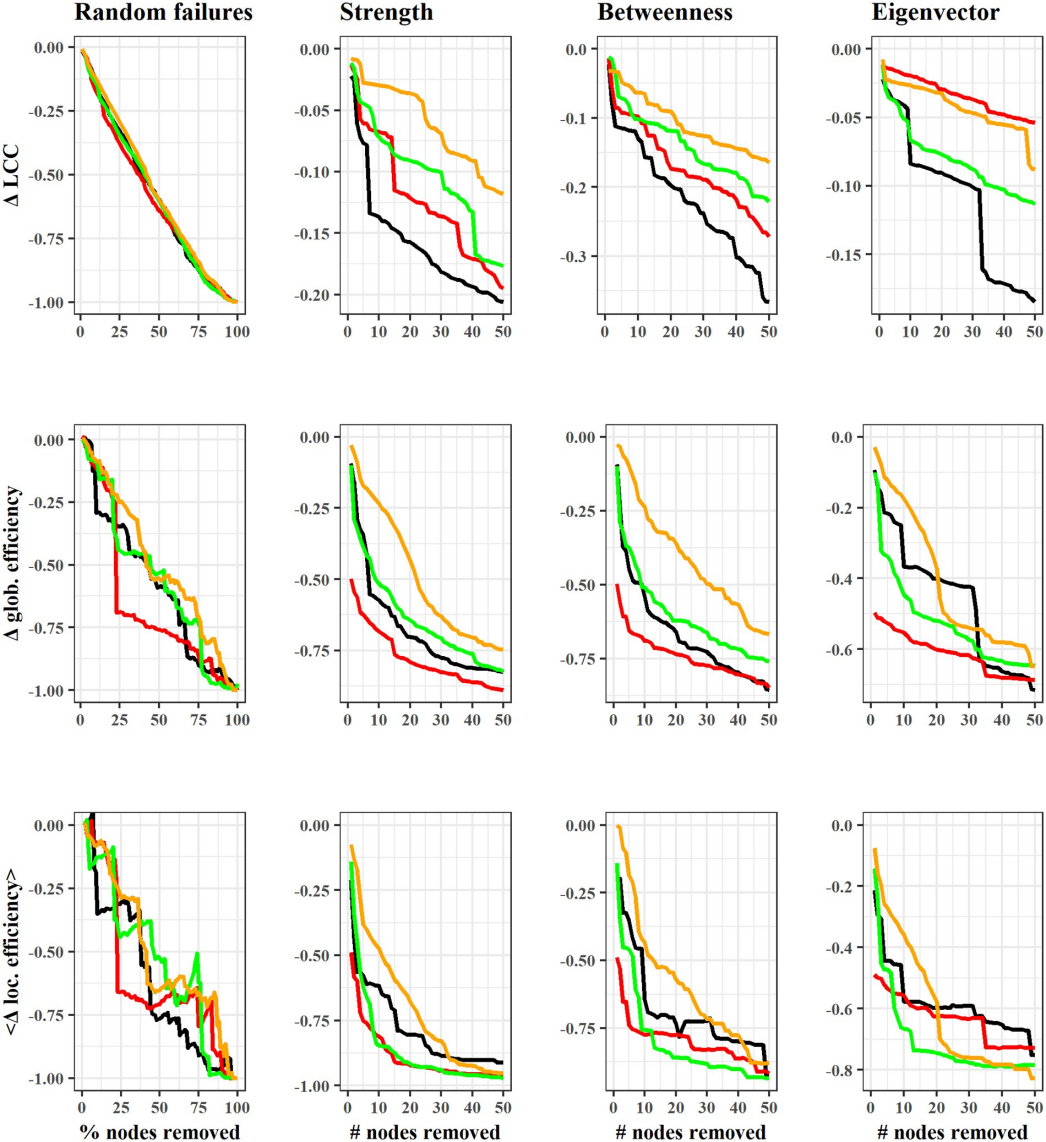
Efficiency drops due to random failures and attacks based on strength, betweenness, and eigenvector centralities. Colors refer to the 12th of: April 2018 (black), July 2018 (red), October 2018 (green), January 2019 (orange). The first column’s x-axis represents the percentage of nodes removed. For the second, third and fourth columns the x-axis is the number of removed nodes. The LN has improved its robustness against random failures and malicious attacks both in terms of local and global efficiency loss.

In our analysis, we consider both random failures and malicious attacks delivered with different strategies based on topological centralities. Fig 4 reports the consequences of removing an increasing number of nodes by showing the impact in terms of the size of the LCC and the global and average local efficiency levels. More generally, the amount of attacked nodes may depend on the size of the LN or be constant assuming that the resources of the attacker(s) are not influenced by the size of the LN itself. Taking into consideration possible attacker’s narrowness of resources, we analyze the loss in efficiency when the attacker is able to remove from one to 50 of the most central nodes. In order to favour the readability of the chart, we plot only four illustrative snapshots (i.e. April, July, and October for year 2018, and January 2019).

As first attack strategy based on topological centrality, we remove the 50 most endowed nodes in the network in terms of strength. We can easily observe the remarkable improvement over time of the LN’s resilience against this type of attack. For instance, in April 2018 the removal of about 10 nodes would have caused the LCC to lose more than 12% of its size, while the removal of the first 50 nodes would have crippled the network by more than 20%. These percentages improve substantially in the configuration corresponding to January 2019, when the potential damage reduces significantly. Similarly, global and local efficiencies appear less affected in more recent periods, although in each period even the removal of some of the most central nodes seems sufficient to affect the efficiency of the system. Interestingly, a strategy based on the removal of most central nodes in terms of betweenness seems to be more effective in severing the LCC. Indeed, both attack strategies based on strength and betweenness centralities have similar effectiveness in damaging the global efficiency levels, although the configuration of January 2019 appears even more resilient than initial configurations under the betweenness attack strategy. Moreover, we assess the robustness of the LN against attacks based on the eigenvector centrality. Compared to the two previous attacks, this strategy appears in general the least effective in terms of LCC’s size reduction, while it is quite similar to the other attack strategies as concerns both global and local efficiency disruption. Finally, the percentage of nodes which can be lost by the LCC after a random failure remains almost stable across the different time snapshots and decreases in a linear fashion with no particular abrupt disconnections. However, the random disappearance of 10% of nodes would have still caused a difference in the global efficiency of more than 25% in the first time snapshot. By contrast, in the last observation this drop would be lower than 12%, thus supporting this improving tendency. Similar patterns emerge for changes in local efficiency levels over time.

Overall, the efficiency of the network seems to be robust against random disruptions. Moreover, despite a remarkable improvement in resilience, the LN can be very much affected by targeted attacks. In particular, a malicious attacker interested in dividing as many nodes as possible from the LCC could adopt a strategy based on the betweenness centrality, or attack the most endowed nodes if he is interested in reducing the global or local efficiency levels of the LN.

### Synchronization

Synchronization is a critical feature for all those systems in which it is desirable to achieve a distributed consensus, i.e., where different participants have to coordinate locally with the aim to increase the global performances of the network [36, 37]. This aspect has been studied in several fields in engineering, such as distributed sensors, parallel and distributed computing, and power grids [37]. For instance, [38] highlight the importance of synchronization for power networks due to their volatile conditions of both the demand and the supply side. Considering the routing system for indirect transactions, we find that the LN presents the same issues since it has both demand and supply sides that are not fixed.

Differently from power transportation systems, LN is a distributed multi-agent infrastructure with no central entity capable of imposing the coordination among its participants. From this perspective, the LN resembles a network made of sensor devices with no central coordinator [39]. Distributed multi-agent networks can reach synchronization by sharing the information they directly have access to. Then, this shared information can be used by each node to rearrange itself in order to increase the efficiency of the entire system. In the LN case, this translates into making the multi-hop routing as efficient as possible. In fact, it is possible to represent the whole LN as an ensemble of multi-state oscillators (here represented by the channels) with three different possible states depending on the balance of the capacity between each pair of nodes, namely: “Open & Balanced”, “Open & Unbalanced” and “Closed”. Practically, a channel can move to “Closed” or “Open & Balanced” from every other state, while the “Open & Unbalanced” state can be reached only from “Open & Balanced”. Although Bitcoin is a decentralized system in which consensus and coordination are enforced by miners using the “Proof of Work” paradigm, the LN has not such feature to enforce coordination among its nodes, thus its synchronization is limited by the ability of its own members to reach a distributed consensus.

Since it is not possible to know the distribution of the capacity in one channel without reaching it directly through the multi-hop path, a more synchronizable topology would help to reduce the effort to collect this kind of information thus reducing the latency during the multi-hop routing of transactions. For instance, [40] measure the propensity of a network towards synchronization by the ratio between the highest and the smallest non-zero value of the Laplacian Matrix’s eigenvalues, namely the *Eigenratio*. In particular, the lower the value of the Eigenratio, the more the network is synchronized and viceversa [41]. [42] associate the Laplacian largest eigenvalue, namely the Laplacian “Spectral Radius”, with the stability of time varying networks. The first smallest non-zero eigenvalue of the Laplacian Matrix (namely, λ_2_ also called “Algebraic Connectivity”) instead assumes non zero value for all connected graphs [43, 44] and governs the rate of convergence of the system towards a distributed consensus [45].

As in other studies about the synchronizability of real world networks [46, 47], we circumscribe our analysis to the Largest Connected Component, which accounts for the near totality of our network participants. The Laplacian of the weighted graph has been found to better describe the coordinability of a system in several real cases, such as biological systems with “prey-predatory” interactions, transportation and neural networks [48]. In our case, the quantity of information a node can learn from its neighbourhood is not affected by the capacity installed on the channels, but only by the presence of a direct link. For these reasons, we opt to analyze the topological coordination of the LN from its un-weighted Laplacian matrix.

Table 3 reports the Eigenratio, the Spectral Radius, and the Algebraic Connectivity for each time snapshot. Our findings show that the LN’s synchronizability after the initial stages has maintained a stable behaviour during most of the sampled period and that the increase of Eigenratios in the last observations mostly relates to the decrease in the Algebraic Connectivity. This relates to a slight degradation in the LN’s capacity to promote coordination among its nodes, which may lead to higher latencies in the transactions routing. As [49] notes, networks with non narrow degree distributions, such as scale-free networks, typically have poorer synchronizability. The sudden increase of the Eigenratios after February 2018 thus seems to suggest the worsening in the likelihood of self-coordination in the network after the initial deployment. Table 3 also indicates that Algebraic Connectivity lies in a plateau at about 0.07 and is steady since March 2018, thus depicting an almost stable connectivity of the underlying LN. However, between November and December 2018, the Eigenratio worsened following a comparable movement by the Algebraic Connectivity. By contrast, after a slight variation in March 2018, the LN’s Spectral Radius remained stable around 1.93, which indicates no particular evolution in the system’s stability.

**Table 3:** LN’s synchronization. The table shows that LN’s topology has evolved into a structure less prone to promote a distributed consensus.

	Feb-18	Mar-18	Apr-18	May-18	Jun-18	Jul-18	Aug-18	Sep-18	Oct-18	Nov-18	Dec-18	Jan-19
*Eigenratio*	8.00	25.36	25.82	25.87	25.94	25.99	26.08	26.15	26.17	26.20	30.08	30.10
*Algebraic Connectivity*	0.222	0.076	0.075	0.074	0.074	0.074	0.074	0.074	0.074	0.074	0.064	0.064
*Spectral Radius*	1.778	1.924	1.925	1.926	1.926	1.926	1.926	1.926	1.926	1.926	1.936	1.936

To sum up, the LN’s topology has evolved during the sampled period into a configuration that seems to worsen the possibility to reach shared consensus. The causes seem to lay in the graph’s connectivity, represented here by the second smallest eigenvalue of the Laplacian matrix.

### Anonymity

Bitcoin has been typically related to anonymity issues. Before becoming a speculative asset, it has achieved fame in part due to its use in the dark market [50]. In transaction networks, it is important to preserve the privacy of the node that broadcasts the message (i.e., the *sender anonymity*), while the privacy of the recipient can be known by other parties. This is true also for the LN, where the receiving node is publicly announced to let the payment route across the network.

Every system provides its users with a certain degree of anonymity, which spans from zero to an absolute privacy, meaning that it is not possible for an attacker to distinguish the effective sender [51]. To preserve the privacy of its nodes, a key role is thus played by the network configuration itself. Some attempts to de-anonymize the components of the network could rely, therefore, upon its topological properties and weakness.

**Fig 5:**
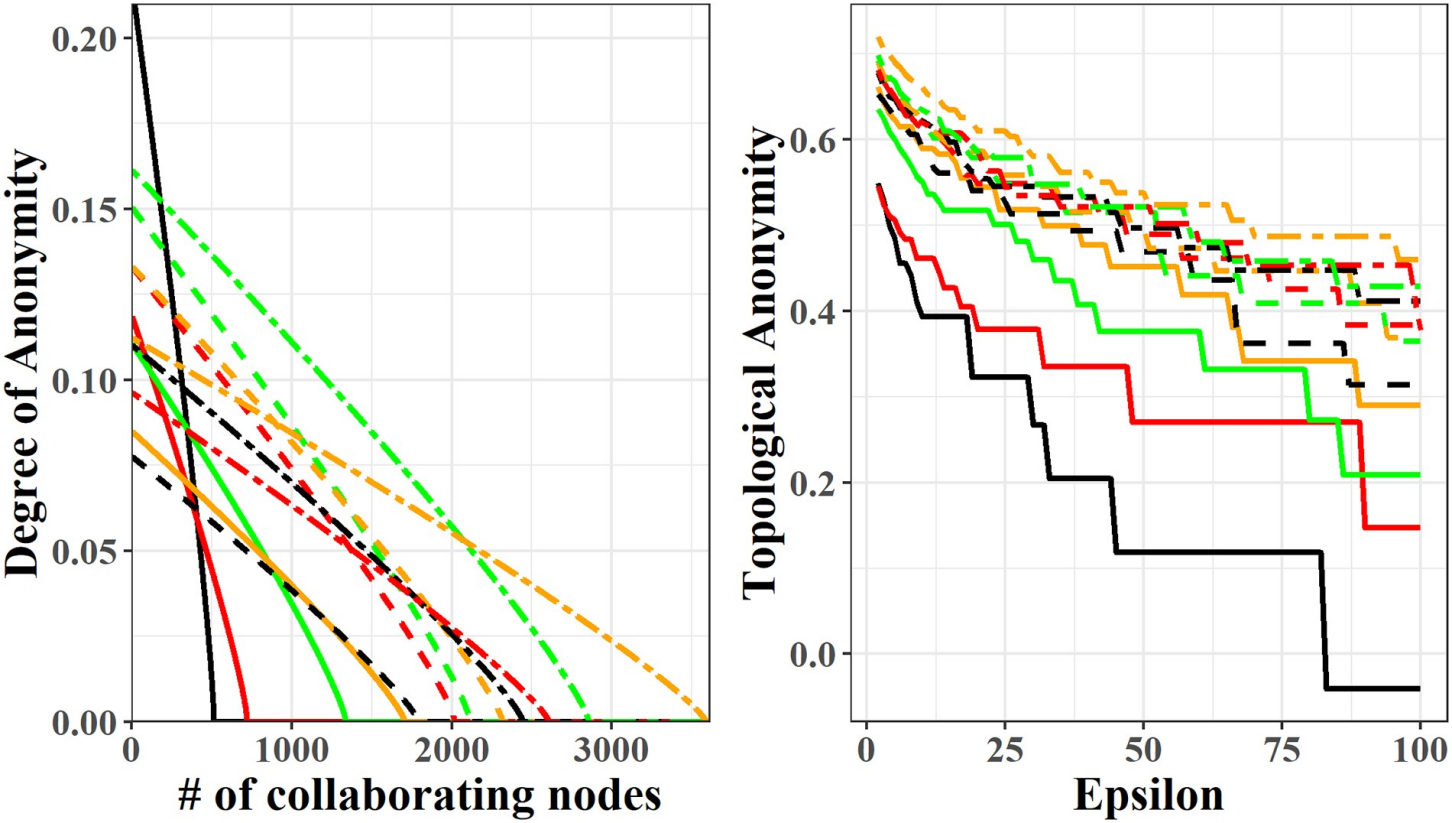
Evolution of LN’s anonymity preservation. Plot on the left refers to the Degree of Anonymity, while plot on the right is for the Topological Anonymity. Continuous lines: Feb-2018 (black), Mar-2018 (red), Apr-2018 (green) and May-2018 (orange). Segmented lines: Jun-2018 (black), Jul-2018 (red), Aug-2018 (green) and Sep-2018 (orange). Double segmented lines: Oct-2018 (black), Nov-2018 (red), Dec-2018 (green) and Jan-2019 (orange).

[52] derived a measure based on entropy for assessing the degree of senders’ anonymity for a certain crowd. Crowds are networks designed to provide privacy to senders by routing the message across a number of other members of the crowd before delivering it to the true receiver. This is precisely what happens with the LN’s multi-hop framework: no intermediate node has the possibility to distinguish if the previous one is just another intermediary or the actual sender. Our analysis relies therefore, on the degree of anonymity that has already been applied to study similar contexts such as cryptocurrencies [53, 54] and the TOR network [55, 56], where the latter is a system with anonymity requirements comparable if not higher than those of cryptocurrencies. More specifically, given a crowd composed by *N* nodes, among which there is one attacker and *C* collaborators, we can describe the entropy of the system as: $H\left(X\right)=\frac{N-p_{f}\left(N-C-1\right)}{N}log_{2}[\frac{N}{N-p_{f}\left(N-C-1\right)}]+p_{f}\frac{N-C-1}{N}log_{2}[\frac{N}{p_{f}}]$, with *p*_*f*_ being the probability of forwarding the transaction to another node. In our case, this probability is approximated by the normalized global efficiencies computed in the section about efficiency. Finally, we obtain the degree of anonymity by dividing the entropy of the system with its maximum defined as *H*_*M*_ = *log*_2_(*N* − *C*). As a result, the degree of anonymity can assume values from 0 to 1. In Fig 5, we analyse the LN’s degree of anonymity during the sampled period. The LN presents a very low degree of anonymity, with only the first time snapshot above 0.2 for a small number of collaborating nodes. Considering the importance that the Bitcoin’s community gives to their own privacy such values should raise concerns.

“Degree of anonymity” is an information theoretic measure used to assess the privacy of a message transmitter node in a network that routes information [57]. [58] propose a measure based on the structure of the network, named *Topological Anonymity* (hereinafter, *TA*), to assess the level of anonymity provided by the structure of the network. This indicator represents a composite measure, derived from the distributions of both degrees and clustering coefficients, which is computed as follows: $TA=\frac{\sum_{i=1}^{\text{max}(\text{deg}(G))}(|D_{i}|*{CC}_{dif_{i}})-\sum_{j=1}^{\;\epsilon -1}|D_{j}|}{N}$, where |*D*_*α*_| the number of nodes with degree *α*, $CC_{dif_{\alpha }}$ the Boolean cluster coefficient that takes a value of 1 if *var*(*CC*(*D*_*α*_)) > 0 and zero otherwise, and parameter *ϵ* that indicates the required level of anonymity of each node. *TA* assumes values ranging from -1, when the network is very prone to node identity disclosure, to +1 which stands for the highest level of privacy preservation. The second plot in Fig 5 reports the *TA* of the LN for different values of the *ϵ* parameter. During the sampled period, the LN has increased its *TA* remarkably going from 0.55 to more than 0.70, for a value of *ϵ* of 2. The improvement is even more visible if we consider larger values of *ϵ*. This means that LN’s topology has evolved into a structure more capable to protect its users’ privacy by prioritising higher levels of anonymity requirements.

Summarizing, LN does not seem to provide a significant anonymity preservation of its users from attacks performed by malicious nodes present on transactions’ paths, while its structure shows a remarkable and increasing strength in protecting the identity of a node from attackers that possess only prior information about topological properties of the target node.

## Discussion

This paper presents a topological analysis of the Bitcoin’s Lightning Network performed during its first year of existence on the mainnet. In this period, the amount of nodes has increased by almost 7 times and the number of available channels simultaneously available by more than 12 times. The value loaded on channels is still negligible if compared with Bitcoin’s $70 billion market cap as of the time of writing, but it is growing rapidly both in total value as well as in the average capacity per channel.

For representative purposes, our analysis is based on consecutive time snapshots which describe the configuration of the LN along its first year of existence. Although there are limitations associated with such an investigation framework, and a need exists for more advanced techniques to study the dynamic evolution of the network, our findings still show how the concerns that the LN would have evolved into a centralized structure were not without basis. The LN seems to be prone to present a structure with highly centralized hubs to whom low degree nodes prefer to attach in order to be able to reach more counterparts without having to establish direct connection with them. Furthermore, we notice that during the sample period, the LN has improved its efficiency both globally and locally due to the increase in capacity installed on its channels. That being said, when compared with other networks, the LN does not seem to have already reached a satisfactory level of efficiency. The LN also appears to be quite resistant against random disruptions. It does not hold however as valiantly as for random failures in case of malicious attacks performed by removing very central nodes with respect to the strength, the eigenvector or the betweenness centralities of its nodes. In addition, the possibility to create the conditions for reaching coordination among its nodes has been shown to be extremely low. Finally, we find contradictory results in the evolution of the anonymity. Studying LN’s ability to preserve its users privacy from an attacker that controls one or more nodes, therefore capable to intercept and study the flow of information within the network, we find that the system poses a weak layer of protection, with low values of degree of anonymity. From a structural point of view, LN is very effective into protecting its nodes’ identities from malicious external observers with only prior knowledge about topological characteristics, such as degree or cluster coefficients, of the target node. Furthermore, this strength of the system is improving over time even for higher privacy requirements.

## Supporting information

S1 Data All relevant data. (CSV)
